# Fluorescence Imaging Topography Scanning System for intraoperative multimodal imaging

**DOI:** 10.1371/journal.pone.0174928

**Published:** 2017-04-24

**Authors:** Tri T. Quang, Hye-Yeong Kim, Forrest Sheng Bao, Francis A. Papay, W. Barry Edwards, Yang Liu

**Affiliations:** 1 Department of Biomedical Engineering, the University of Akron, Akron, Ohio, United States of America; 2 Department of Radiology, University of Pittsburgh, Pittsburgh, Pennsylvania, United States of America; 3 Department of Electrical and Computer Engineering, the University of Akron, Akron, Ohio, United States of America; 4 Dermatology and Plastic Surgery Institute, Cleveland Clinic, Cleveland, Ohio, United States of America; Tufts University, UNITED STATES

## Abstract

Fluorescence imaging is a powerful technique with diverse applications in intraoperative settings. Visualization of three dimensional (3D) structures and depth assessment of lesions, however, are oftentimes limited in planar fluorescence imaging systems. In this study, a novel Fluorescence Imaging Topography Scanning (FITS) system has been developed, which offers color reflectance imaging, fluorescence imaging and surface topography scanning capabilities. The system is compact and portable, and thus suitable for deployment in the operating room without disturbing the surgical flow. For system performance, parameters including near infrared fluorescence detection limit, contrast transfer functions and topography depth resolution were characterized. The developed system was tested in chicken tissues ex vivo with simulated tumors for intraoperative imaging. We subsequently conducted in vivo multimodal imaging of sentinel lymph nodes in mice using FITS and PET/CT. The PET/CT/optical multimodal images were co-registered and conveniently presented to users to guide surgeries. Our results show that the developed system can facilitate multimodal intraoperative imaging.

## Introduction

Multimodal imaging is a promising strategy to overcome limitations of medical imaging technologies by combining strengths of individual modalities. Clinically, multimodal imaging modalities such as positron emission tomography and x-ray computed tomography (PET/CT) and single photon emission computed tomography and x-ray computed tomography (SPECT/CT) are used to capture and present both functional and anatomical data to physicians, facilitating clinical decision making. However, their utility in intraoperative settings is limited due to their high cost and the extended scanning time [1–6]. Moreover, these imaging modalities lack real time update, which is crucial for guiding surgeries.

Optical imaging, on the other hand, enables real time imaging and thus represents a promising technology for intraoperative guidance. Compared with visible light, near-infrared (NIR) spectral region (650–900 nm) offers deeper penetration, reduced tissue scattering and absorption, and minimal autofluorescence, making the NIR window ideal for clinical applications [7, 8]. In recent years, fluorescence imaging has attracted significant attention in the fields of intraoperative imaging, image-guided therapy and surgery [1–3, 9–15]. Since planar fluorescence imaging cannot provide depth or size information, several multimodal imaging approaches in which tomographic fluorescence imaging is integrated with other imaging modalities have been proposed for 3D visualization. For instance, dual-modality imaging systems consisting of fluorescence molecular tomography (FMT) and CT have been developed and applied to various medical applications [16–19]. A bimodal imaging system that incorporates an FMT system into MRI was introduced for a small animal simultaneous imaging acquisition [20]. In combination with nuclear imaging, Li et al. integrated fluorescence tomography with PET for studying multimodal imaging probes and complementary pairs of radiotracers [21]. van Oosterom et al., on the other hand, integrated bioluminescent and fluorescence imaging into SPECT to provide high-resolution and highly quantitative SPECT information about tracer kinetics [22]. These systems are excellent for research purposes but not optimized for intraoperative guidance due to their limitations in scan time as the FMT technology needs relatively long period of time for image acquisition and reconstruction. The resolution is also limited, hindering its use in the surgical applications such as tumor margin imaging.

To overcome these challenges, we have developed a novel Fluorescence Imaging Topography Scanning (FITS) system. The system can capture intraoperative fluorescence, color reflectance and surface topography. Several experiments were performed on small animal models to validate the capabilities of the system in providing multimodal imaging, navigation and guidance for surgery. We also performed multimodal imaging of the sentinel lymph nodes (SLN) using FITS and PET/CT in vivo. The preoperative PET/CT data co-registered with intraoperative FITS data can provide depth information and multimodal image guidance for surgery.

## Materials and methods

### Fluorescence Imaging Topography Scanning system

**Fig 1:**
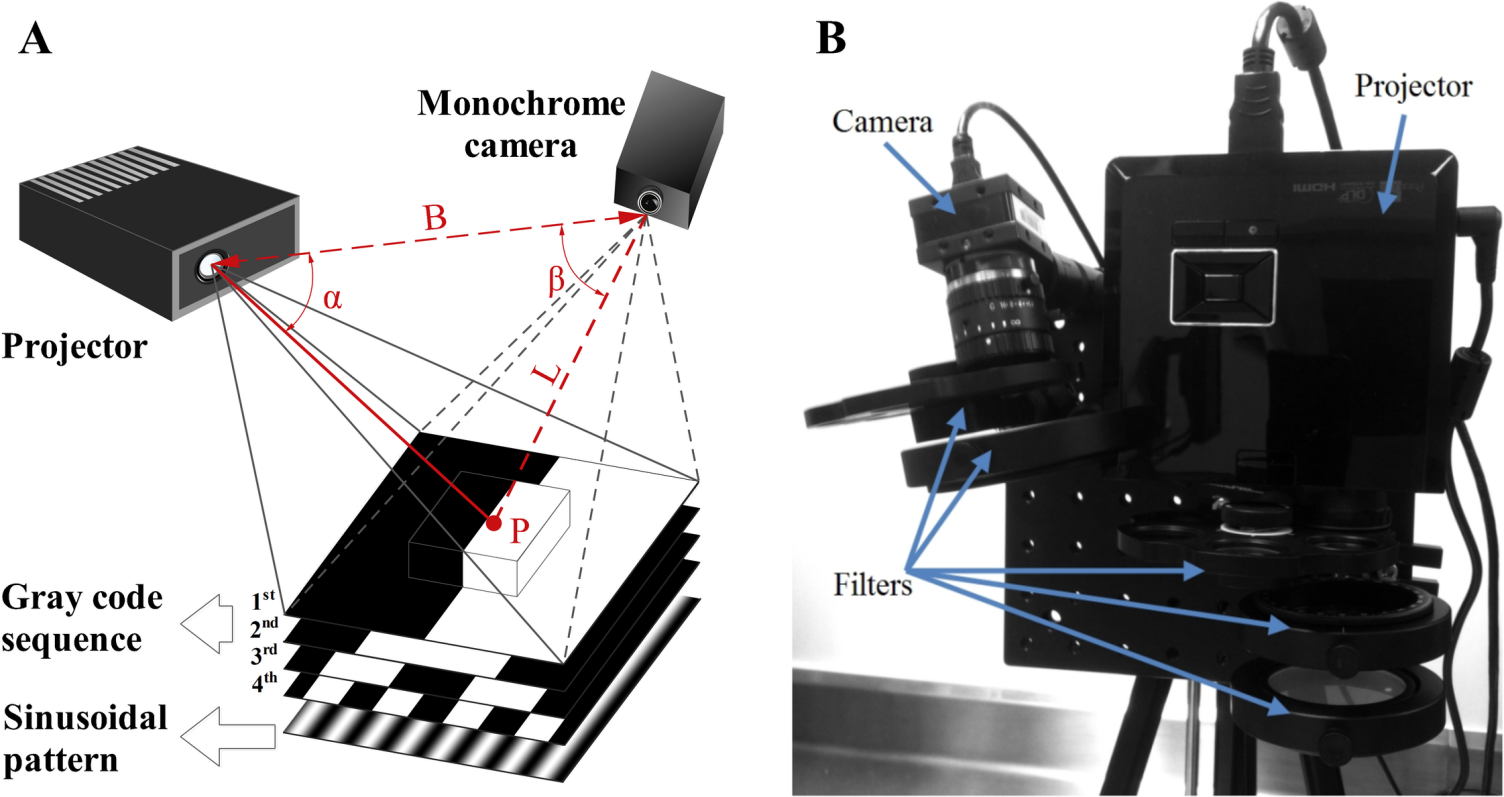
Fluorescence Imaging Topography Scanning (FITS) system. (A) A schematic illustration of 3D topography scanning with sequential gray code projections. (B) Top view of the prototype FITS system.

The FITS system was developed to perform topography scanning by applying coded structured-light imaging [23]. A typical structured-light 3D surface imaging system includes a projector and a camera which are geometrically calibrated as shown in Fig 1A. In principle, the distortion of the projected structured-light patterns imaged by the camera in comparison with the undistorted projection patterns is computed to extract the 3D coordinates of objects. We implemented a combination of the gray-code coding and phase shift techniques to achieve surface structures based on a temporal sequence of black and white stripe and sinusoidal patterns [23]. Gray codes originally proposed by Inokuchi et al. in 1984 [24] and phase-shifted patterns with a sinusoidal intensity modulation are illustrated in Fig 1A. The length of the codeword is given by 2^n^ bits, where n is the total number of projected patterns. Particularly, a unique code refers to each point on the surface of the object. A large number of sequential gray-code patterns are needed to achieve a high spatial resolution. The combination of the gray-code coding and phase shift techniques increases the spatial resolution as the phase shift technique offers sub-pixel resolution beyond the number of gray codes projected [23]. After sequential gray-code and sinusoidal pattern projections of both horizontal and vertical stripes, the column and row lines form a full frame of 3D images. Coding procedures assign accurate correspondences for each point in the projector, projection patterns and detector pixels. The triangulation principle as Eq 1 is then applied to compute the correspondences for a 3D point cloud as P_i_ = (x_i_,y_i_,z_i_,I_i_), i = 1:N, where N is the total points in the surface, and I_i_ is the value of the i^th^ surface point.

$$L=B\frac{\sin(\alpha )}{\sin(\alpha +\beta )}$$ (1)

The geometric relationship between the detector, a structured-light projector, and an object surface point is depicted in Fig 1A.

All components of the FITS prototype as shown in Fig 1B including a projector (LED K11, Acer), a monochrome camera (DMK 42BUC03, The Imaging Source with Computar 12mm lens) and filters are assembled on a compact optical breadboard which can be mounted on a standard tripod for easy deployment in clinical settings. The projector and camera are connected to a laptop computer. The projector is also employed as the light source for fluorescence imaging. A short-pass filter (775 nm) (Edmund Optics, NJ) and a band-pass filter (832 ± 37 nm) (Edmund Optics, NJ) are attached to the projector and the camera for provision of excitation and filtration of emission wavelengths of ICG, respectively. The system was calibrated for an optimal working distance range from 268 mm to 467 mm. Camera exposure time was set at 100 milliseconds for all measurements. As developed, the FITS system offers intraoperative surface topography scanning, color reflectance and fluorescence imaging. The prototype is a lightweight (3.6 kg) and compact in design (23x23x23 cm) and thus can be easily transported and deployed in the operating room.

### System characterization

NIR fluorescence detection limit, contrast transfer functions and topography depth resolution were investigated to evaluate system performance. Indocyanine green (Sigma Aldrich, MO) dissolved in a dimethyl sulfoxide (DMSO) solvent (Sigma Aldrich, MO) was prepared for studies and testing the system. A set of diluted fluorescence solutions with various ICG concentrations including 10 nM, 20 nM, 55 nM, 95 nM, 185 nM, and 370 nM was used to characterize ICG sensitivity. For each concentration, three 0.3 mL circular cylinder capsules of ICG solution and a DMSO reference capsule were placed on chicken tissue and then imaged by the system at two working distances (300 mm and 400 mm). The detection limits for ICG were determined afterwards.

The 1951 USAF resolution test chart (Edmund Optics, NJ) was utilized for contrast characterization. Images of the chart captured at five working distances of the camera including 300 mm, 350 mm, 400 mm, 450 mm, and 500 mm were analyzed. Contrast transfer functions (CTF) as the correlation between contrast and spatial frequency were then quantified. For depth resolution, two thin plates in which one of them was placed at different depths to the other were scanned at 300 mm from the system. To further characterize the FITS prototype performance, two parallel plastic tubes containing 200 nM ICG were laterally separated by distances of 0.1 mm, 0.25 mm, 0.5 mm, 0.75 mm and 1 mm, and observed at different working distances to determine fluorescence resolution of the system.

### Ex vivo image-guided surgeries in chicken

In this study, two 0.3 mL circular cylinder capsules were implanted under the chicken leg skin, simulating protruding tumors. One capsule containing ICG solution is used to simulate a malignant tumor, and the other capsule containing deionized water was used as the control. In another experiment, the ICG was directly injected into the chicken tissues, in which the skin was removed, to simulate infiltrating tumors with a diffusive margin. The FITS system was used to identify the simulated malignant tumor and fluorescence-labeled tissues in the chicken and subsequently conduct image-guided surgeries ex vivo.

The imaging procedure comprises topography scanning, color reflectance, fluorescence imaging and image processing. The chicken leg was placed within the calibrated working distance range for optimal intraoperative imaging. First, the system was adjusted to obtain the best focus and scanned for surface structures. After obtaining a mesh topography, red, green and blue light were sequentially projected on the object and captured by the detector, which were used to synthesize a color reflectance image based on the division-of-time technique. The fluorescence-labeled targets were subsequently illuminated by the light source with excitation filter in the light path, and the detector captured the images with the NIR emission filter in the light path. The fluorescence image was overlaid with the true-color reflectance image to create a composite image where fluorescence information was pseudocolored in green. Finally, the fluorescence-reflectance composite image was mapped and registered to the topography.

### In vivo multimodal imaging of mouse SLNs with PET/CT

Sentinel lymph node biopsy is the standard staging technique for breast cancers and melanomas. In clinical settings, radionuclides and blue dye are often used together for lymph node mapping. NIR mapping of SLNs have also been conducted in recent years [9–15]. In this study, we tested the FITS system in multimodal imaging of SLNs with both radionuclides and ICG.

#### Radiotracer synthesis

Gallium-68 (^68^Ga) production: All chemicals were purchased from Sigma-Aldrich Chemical Co. (St. Louis, MO), unless otherwise stated: Aqueous solutions were prepared using ultrapure water (resistivity, 18 MΩ). ^68^Ga (*t*_*1/2*_ = 68 min) was produced from a ^68^Ge/^68^Ga generator (IGG100, Eckert and Ziegler, Berlin, Germany) and purified using a cation exchange column (Strata-X-C, Phenomenex). ~1 mL of ^68^Ga (650 MBq) was obtained in 0.02 M HCl and 98% acetone.

^68^Ga labeling: NODAGA-c(RGDyK) (4.0 nmol, ABX GmbH: advanced biochemical compounds, Radeberg, Germany) was mixed with ^68^Ga (~90 MBq) in ammonium acetate buffer (100–150 μL, 0.1 M, pH 4.0) and incubated for 15 min at room temperature. Radiochemical purity was determined using radio reversed-phase high-performance liquid chromatography (RP-HPLC). The radiotracer solution was diluted with sterile 0.9% NaCl for animal studies.

#### SLN biopsy guided by FITS-aided multimodal fluorescence/PET/CT guidance

All animal studies were conducted according to the protocols approved by the Animal Care and Use Committee at the University of Pittsburgh. During radiotracer injection and imaging, the mice were anesthetized with 2% isoflurane. ^68^Ga-NODAGA-c(RGDyK) (5–7 MBq, ~100 μL) and 10 μL of ICG in saline solution (7.82 nanomoles ICG) was co-injected in the right forepaw of ICR/CD-1 mouse (Charles River Laboratories Inc., Wilmington, MA). Gentle clipping and cream depilation removed the hair overlaying the region of interest. For animal studies, the mice were anesthetized with ketamine and xylazine cocktail (80 mg/kg and 13 mg/kg, respectively, intraperitoneal injection), subsequently attached and fixed on platforms with markers during experiments to maintain their geometrical positions for all imaging modalities. The mice were imaged using an Inveon Small Animal PET/CT (Siemens Molecular Imaging, Knoxville, TN) and sequentially imaged with the FITS system. The subcutaneous axillary region was exposed by incising and retracting the overlaying skin under image-guidance of FITS. Accordingly, SLNs were identified and resected. After resection, surgical sites were inspected thoroughly with the FITS system. SLNs were examined with the system ex vivo. Excised tissues were embedded in Tissue-Tek OCT medium (Sakura Finetek, Torrance, CA, USA) and stored at -80°C. Standard histology was performed to confirm the in vivo findings. The mice were sacrificed immediately after imaging experiments by IP injection of 150 mg/kg sodium pentobarbital (Somnosal, MedVet, Worthington, OH, USA).

#### Image co-registration

We used feature-based image registration, in which image features such as edges, corners, lines, curves, regions, templates, and patches are utilized to establish point-by-point correspondences between images from different imaging modalities [25]. The FITS data containing topography/fluorescence/reflectance and the PET/CT images were preprocessed separately before co-registration. Surface topography was mapped to fluorescence-reflectance composite image from the FITS system, using perspective correct mapping shown in Eq 2.

$$u_{\alpha }=\frac{(1-\alpha )\frac{u_{0}}{z_{0}}+\alpha \frac{u_{1}}{z_{1}}}{(1-\alpha )\frac{1}{z_{0}}+\alpha \frac{1}{z_{1}}}=\frac{z_{1}u_{0}-\alpha u_{0}z_{1}+\alpha u_{1}z_{0}}{z_{1}-\alpha z_{1}+\alpha z_{0}}$$ (2)

Where u_α_ is the image coordinate between two endpoints u_0_ and u_1_. The perspective correct mapping interpolates after dividing by depth z, then uses its interpolated reciprocal to recover the correct coordinate. After mapping the topography/fluorescence/color data was imported in MeshLab (Visual Computing Lab, Pisa, Italy) and manually cropped to remove unnecessary faces and vertices of the model and meshes outside the regions of interest. DICOM data from the PET/CT was imported in ImageJ (NIH, USA) and manually segmented based on intensity to extract information of different tissue types. The PET/CT data were saved in OBJ files and then imported and rescaled in MeshLab. We employed the alignment tools in MeshLab to co-register PET and CT data into the FITS data [26]. Common points between the FITS and PET/CT models were manually selected based on features of the target, the markers and the platform and used to establish point-by-point correspondences. The FITS and PET/CT meshes were roughly aligned based on the correspondences and subsequently refined with ICP (Iterative Closest Point) and global alignment. The final transformation matrix was defined and applied to precisely register the PET and CT into the FITS models. Transparency of the models in the alpha composition was adjusted to enable visualization of multimodal information in registered images for intuitive visualization.

## Results

### System characterization

**Fig 2:**
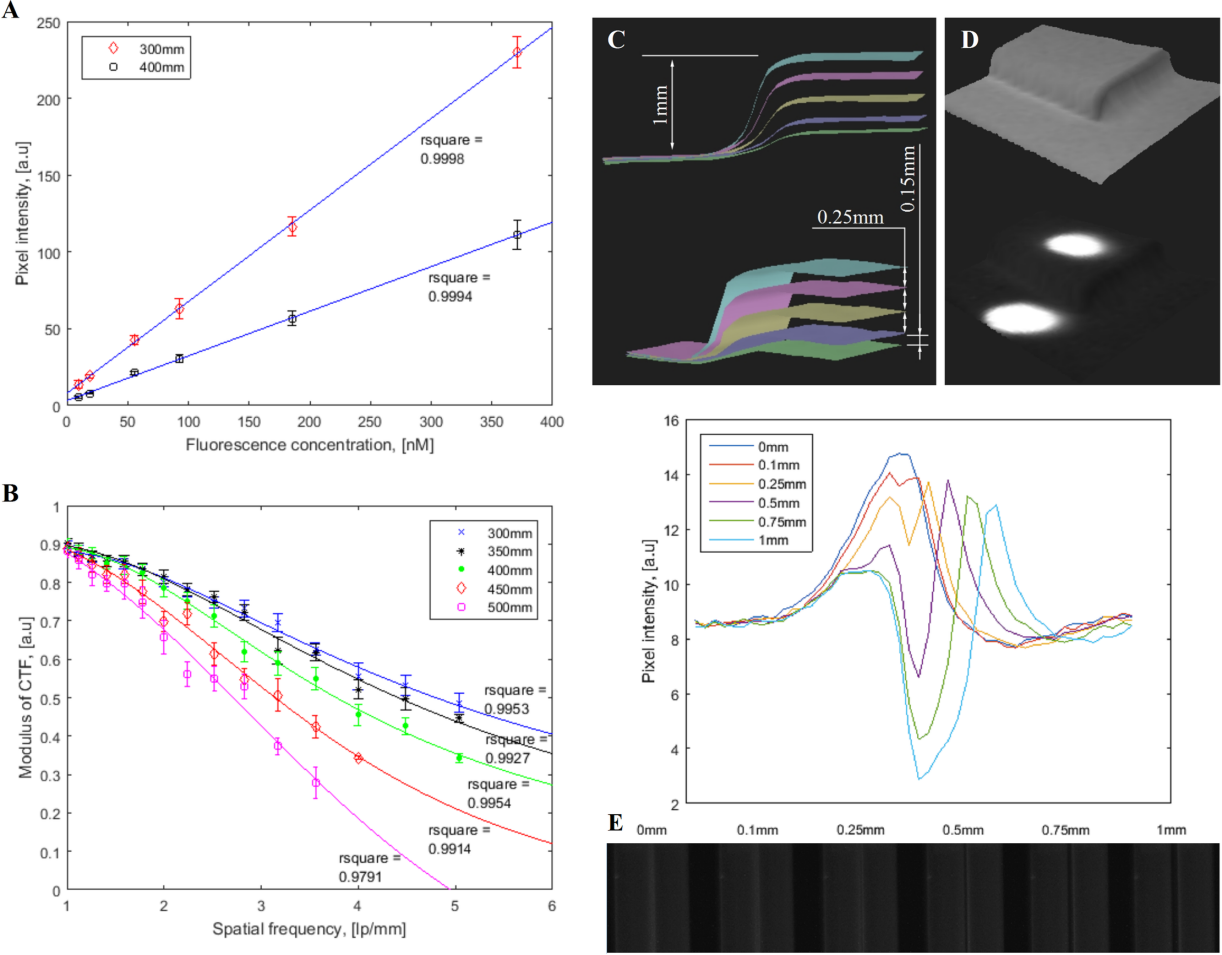
System characterization. (A) Indocyanine green fluorescence detection sensitivity. (B) Contrast transfer functions at various working distances. (C) Topography depth resolution. (D) Resolve fluorescence objects at different depths, where upper fluorescence target is placed on a plane 1 mm higher than the lower target. (E) Lateral resolution with fluorescent dye filled plastic tubes.

The correlation between fluorescence pixel intensity and ICG concentration presented good linearity within various working distances, as shown in Fig 2A. All fitting lines fit the data points with high R-squared values (> 0.99). As ICG concentration increased, fluorescence signal intensity became saturated faster at a shorter working distance. The experimental results showed that the system can detect an ICG concentration down to 10 nM at a signal-to-background ratio of at least 2.

The system was able to reach a spatial resolution of 5 line pairs per millimeter with 35% contrast at 400 mm working distance (Fig 2B). A better contrast was obtained at closer working distances such as 300 mm and 350 mm, as expected. The contrast at 500 mm was more rapidly degraded than the others due to the further working distance, indicating upper limit of optimal working distance range. Various depths including 0.1 mm, 0.25 mm, 0.5 mm, 0.75 mm and 1 mm were investigated to determine topography depth resolution as demonstrated in Fig 2C. The minimum difference in depth that the system can identify was 0.1 mm. Resolution of fluorescent targets on different heights were also achieved to demonstrate the utility of topography detection (Fig 2D). In addition, two side-by-side fluorescent dye filled tubes laterally separated by various distances were imaged to determine lateral resolution as illustrated in Fig 2E. An average intensity profile across two tubes was plotted for each image. Two peaks in the profile plots indicate a distinguishable separation between two fluorescent tubes. As a result, the FITS prototype was able to differentiate a minimum lateral distance of 0.1 mm between two fluorescent tubes at 300 mm working distance. The ICG lateral resolution decreased as working distance increased, as expected.

The aforementioned characterization results showed that the FITS prototype had a 0.1 mm depth resolution for topography and a lateral resolution of 0.1 mm. It offered a fluorescence detection limit of 10 nM and good linearity across the different fluorophore concentrations.

### Ex vivo image-guided surgeries

**Fig 3:**
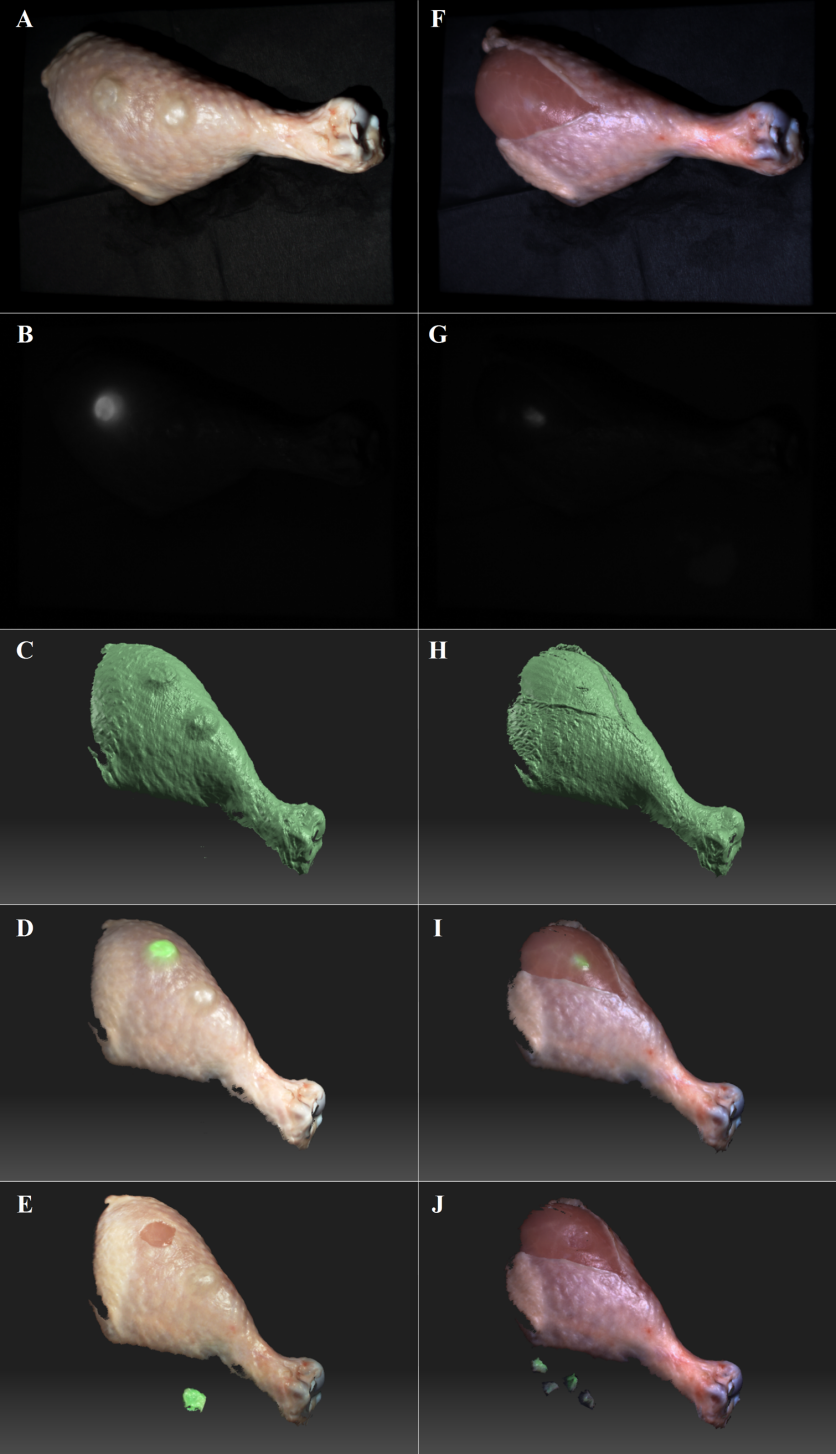
Ex vivo image-guided surgeries in chicken. (A-D) Color reflectance, NIR fluorescence, surface topography, and surface topography image registered with the fluorescence/reflectance, of simulated protruding tumors, respectively. (F-I) Color reflectance, NIR fluorescence, surface topography, and surface topography image registered with the fluorescence/reflectance, of a simulated diffusely infiltrating tumor, respectively. (E) & (J) are surface topography images registered with the fluorescence/reflectance, of the surgical scene after the image-guided resections.

Imaging of chicken tissues was successfully performed using the FITS system. The prototype acquired topography, color reflectance and NIR fluorescence of chicken tissues and facilitated ex vivo image-guided surgeries (Fig 3). Color reflectance and surface topography images of the chicken leg are shown in Fig 3A and 3C, respectively. The fluorescence-labeled protruding tumor was imaged by fluorescence imaging as illustrated in Fig 3B. Topography data registered with color reflectance and fluorescence images provided both anatomical and functional information to guide surgery (Fig 3D). In another study, Fig 3F–3I shows color reflectance image, NIR fluorescence image, surface topography, and surface topography registered with color reflectance and NIR fluorescence of a simulated diffusely infiltrating tumor, respectively. With the image guidance shown in Fig 3I, the user was able to conveniently identify and locate the simulated cancerous tissues labeled by ICG. The prototype also provided real-time fluorescence imaging for facilitation of image-guided surgeries. The FITS images (Fig 3D and 3I) offered a better perception of shape and depth of the organs/tissues than conventional planer 2D imaging systems (Fig 3B and 3G), facilitating image-guided surgical resections. The simulated malignant protruding and diffusely infiltrating tumors were completely removed from the chicken legs as shown in Fig 3E and 3J, respectively.

### In vivo multimodal imaging of mouse SLNs with PET/CT

**Fig 4:**
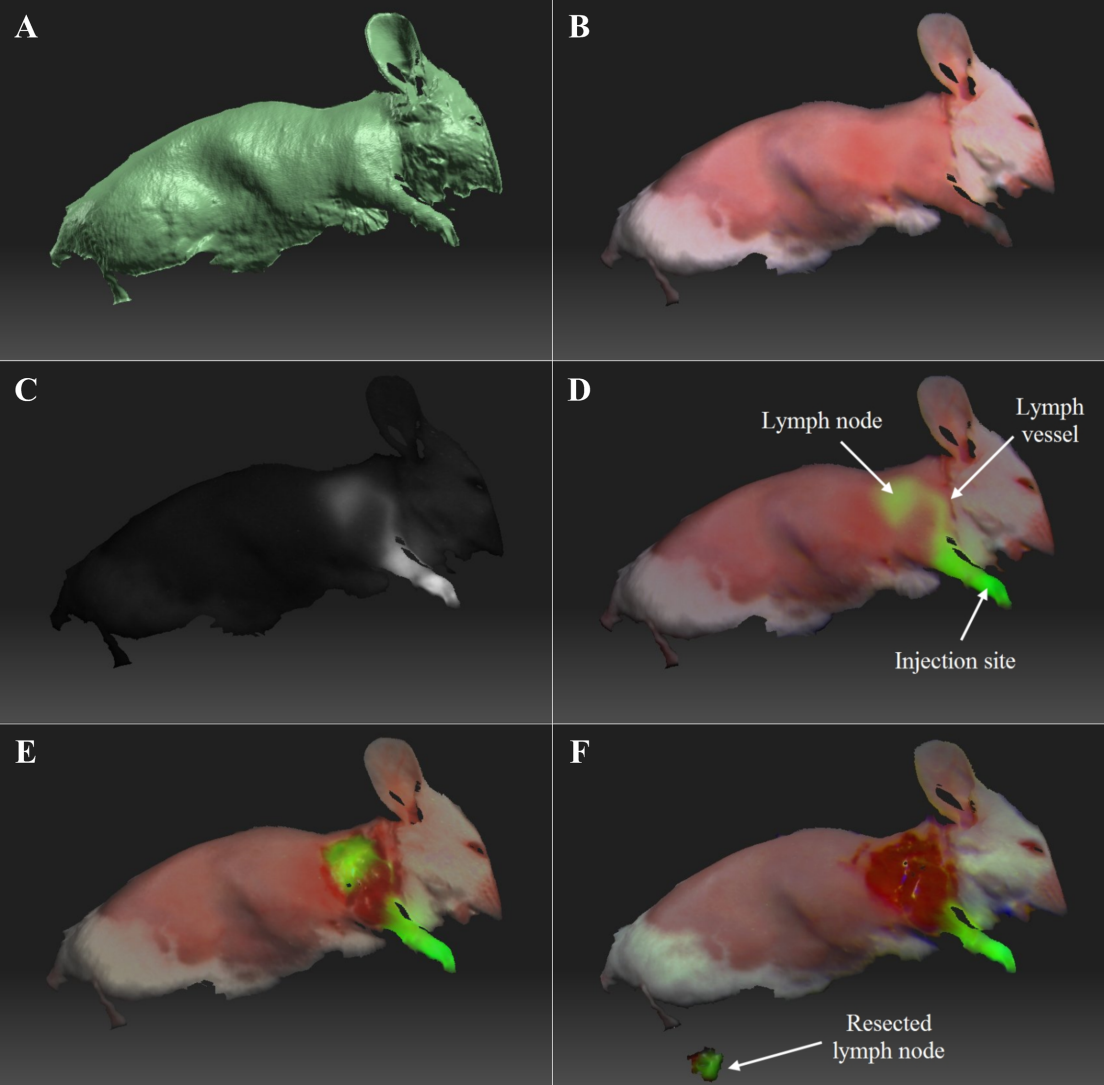
Ex vivo image-guided surgeries in mice. (A) Surface topography. (B) Surface topography registered with color reflectance. (C) Surface topography registered with NIR fluorescence. (D) Surface topography registered with the reflectance and fluorescence, of the mouse prior to the surgery. (E) Surface topography registered with the reflectance and fluorescence, of the mouse with lymph node exposed. (F) Surface topography registered with the reflectance and fluorescence, of the mouse after the lymph node was removed.

For in vivo SLN mapping study, intraoperative fluorescence imaging, color reflectance imaging, topography scanning and image-guided surgeries in mice were conducted using the FITS system (Fig 4). The lymph vessel (diameter < 0.3 mm) connecting the tracer injection site (forepaw) and lymph node were clearly imaged by the FITS prototype. The FITS image in which surface topography was registered with the color reflectance and fluorescence information is shown in Fig 4D. An average signal-to-background ratio of the lymph nodes (n = 3) was 6.09 ± 0.6 ≥ the minimal SBR of 1.1 for positive identification of NIR fluorescence labeled SLNs by in vivo [27]. The prototype successfully identified the lymph nodes, and surgical resections were then facilitated by the guidance of real-time fluorescence imaging aided by the FITS system. Fig 4E and 4F illustrate exposed lymph nodes after overlaying skin removal and complete lymph node resection, respectively. The lymph nodes removed were subsequently imaged ex vivo (Fig 4F).

**Fig 5:**
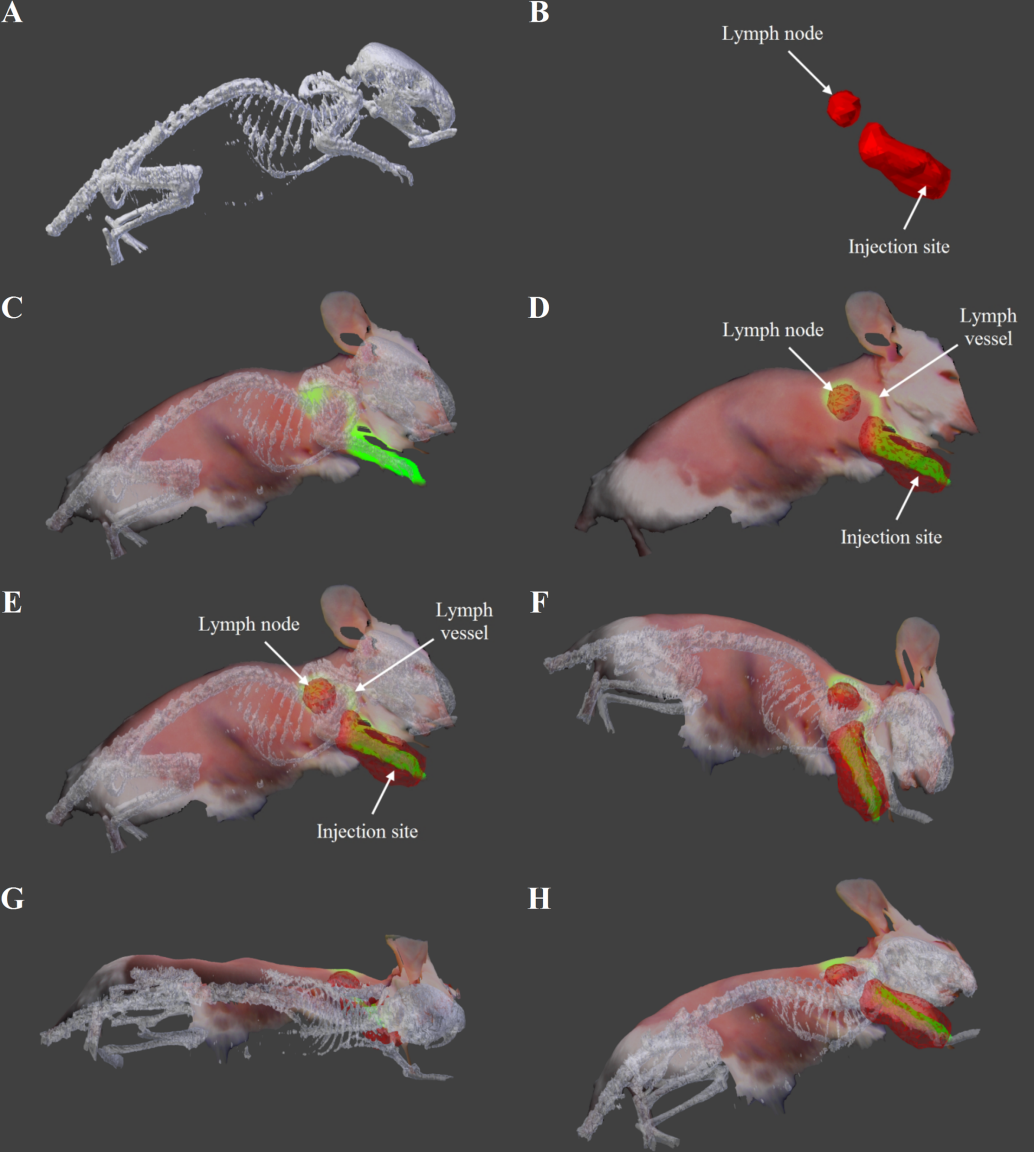
Multimodal imaging of mouse SLNs with PET/CT & FITS system. (A) CT image. (B) PET image. (C) Composite FITS image (topography/reflectance/fluorescence) co-registered with CT data. (D) Composite FITS image (topography/reflectance/fluorescence) co-registered with PET data. (E-H) Composite FITS image (topography/reflectance/fluorescence) co-registered with PET/CT at different viewpoints.

The PET/CT data were co-registered with the FITS data to present final results for in vivo multimodal mouse lymph node imaging (Fig 5). Preprocessed CT and PET data before co-registration are shown in Fig 5A and 5B, respectively. The FITS images co-registered with CT and PET data separately are exhibited in Fig 5C and 5D. The co-registered images shown in Fig 5E–5H contain structural and functional information from all of the imaging modalities. The alignment of PET/CT and FITS data was done in Meshlab with a global error bound of 0.0010 for a satisfactory co-registration [28]. CT data provided anatomical information while color reflectance and topography provided surface profile. PET and fluorescence data co-located at the lymph nodes, which were later confirmed by standard histology. Furthermore, the multimodal data were presented in a 3D model that can be rotated to offer convenient inspection of the mice at different perspective to determine the location of the lymph nodes. The rotated images can convey complementary anatomical and depth information to clinicians for better image guidance and surgical planning. We have created a video showing 360° rotations of the models around the axes to illustrate the dynamic visualization of the objects at various viewpoints with depth perception (Supporting Information: S1 Video).

## Discussion

In this study, we have demonstrated the concept of FITS system, the instrumentation design and the feasibility of using the system for intraoperative multimodal imaging and surgical navigation. We have characterized and demonstrated the capabilities of the system in providing multimodal imaging, surgical navigation and image-guided surgeries ex vivo and in vivo in small animals. In ex vivo study, we have shown that color reflectance, NIR fluorescence and surface topography shows complementary information to the user and the reflectance-fluorescence-topography composite images provide a convenient way for image guidance and adaptive medical data visualization. The FITS system offered high resolution to detect the lesions. In the study of in vivo mouse lymph node mapping, the FITS prototype allowed 3D visualization of a sub-millimeter diameter lymph vessel and the entire draining lymphatic system from the paw to the lymph node.

Powered by the multimodal imaging of the FITS/PET/CT, the advantages of individual modalities were combined while the limitations were mitigated. The topography data captured by the FITS system facilitates co-registration between PET/CT data and fluorescence/reflectance data. Under multimodal image guidance based on PET/CT/FITS, both preoperative and intraoperative image data were presented to users concurrently. The multimodal imaging data facilitates both surgical planning and intraoperative imaging. The promise of using the FITS for guidance of various surgical procedures has been shown.

With the FITS system, the surgeon will have the opportunity to interrogate the status of lesions based on multimodal image data. Both preoperative image and intraoperative image data will be presented to surgeons conveniently for clinical decision support. The difficulties in correlating PET/CT with intraoperative fluorescence data were greatly reduced. In addition, multiscale imaging was achieved, where both whole-body anatomical reference and high-resolution organ level functional image data can be presented to the user for adaptive visualization and inspection. While robotic surgeries are a promising platform for many surgical procedures, the majority of complex surgical procedures are still open surgeries. The FITS systems expand the reach of the intraoperative multimodal image-guidance to all surgeons. Also, commercial robotic surgery systems such as the Da Vinci^TM^ lack the capability to register intraoperative fluorescence images to preoperative PET/CT data to provide integrated surgical navigation. The FITS system adds clinical values for intraoperative decision support while reducing the need for coordinating image data from multiple sources.

Limitations and Future Work: The FITS system discussed herein is still a prototype, which needs further development and optimization for future clinical translation. The experiments presented in the manuscript (e.g. in vivo imaging of mice) refer to models where the subjects present minor movements. In clinical medicine, motion artifacts may cause errors. We will overcome the motion artifact in the future by accelerating the scan time and further development of feature-based image registration algorithm. In the future, we will investigate real time image reconstruction. The image registration algorithm also needs to be further developed to enhance its usability by clinicians in surgical settings. The graphic user interface (GUI) will be further optimized to facilitate the rapid adoption of users. We will also explore the potential of autofluorescence imaging in the visible spectra to minimize the potential toxicity induced by exogenous contrast agents.

The FITS system is a promising technology that can be broadly applied in various types of surgeries including oncologic surgeries, plastic surgeries and other types of open surgery. In addition to SLN mapping, we will investigate other surgical applications in the future. It is a non-contact imaging system, which is safe to use and has translational potential. The system can be deployed in the operating room, fully compatible with the existing surgical workflow.

## Conclusions

We have developed a novel Fluorescence Imaging Topography Scanning (FITS) system for image-guided intraoperative interventions and multimodal imaging. The FITS prototype is small and can be deployed in the operating room fully compatible with existing surgical workflow. We have validated the prototype FITS system in sentinel lymph node mapping in vivo in a mouse model. The system has demonstrated great potential for intraoperative multimodal imaging and surgical navigation.

## Supporting information

S1 Video Supporting video of multimodal imaging and surgical navigation using the Fluorescence Imaging Topography Scanning system and PET/CT. The video shows 360° rotations of the models around the axes was created to illustrate the dynamic visualization of the objects at various viewpoints.
(AVI)
